# Developing an informational tool for ethical engagement in medical tourism

**DOI:** 10.1186/s13010-017-0045-9

**Published:** 2017-08-25

**Authors:** Krystyna Adams, Jeremy Snyder, Valorie A. Crooks, Rory Johnston

**Affiliations:** 1 Faculty of Health Sciences, Simon Fraser University, Blusson Hall, 8888 University Drive, Burnaby, BC Canada; 2 Department of Geography, Simon Fraser University, Robert C. Brown Building, 8888 University Drive, Burnaby, BC Canada

**Keywords:** Medical tourism, International medical travel, Guideline, Ethics, Equity

## Abstract

**Background:**

Medical tourism, the practice of persons intentionally travelling across international boundaries to access medical care, has drawn increasing attention from researchers, particularly in relation to potential ethical concerns of this practice. Researchers have expressed concern for potential negative impacts to individual safety, public health within both countries of origin for medical tourists and destination countries, and global health equity. However, these ethical concerns are not discussed within the sources of information commonly provided to medical tourists, and as such, medical tourists may not be aware of these concerns when engaging in medical tourism. This paper describes the methodology utilized to develop an information sheet intended to be disseminated to Canadian medical tourists to encourage contemplation and further public discussion of the ethical concerns in medical tourism.

**Methods:**

The methodology for developing the information sheet drew on an iterative process to consider stakeholder feedback on the content and use of the information sheet as it might inform prospective medical tourists’ decision making. This methodology includes a literature review as well as formative research with Canadian public health professionals and former medical tourists.

**Results:**

The final information sheet underwent numerous revisions throughout the formative research process according to feedback from medical tourism stakeholders. These revisions focused primarily on making the information sheet concise with points that encourage individuals considering travelling for medical tourism to do further research regarding their safety both within the destination country, while travelling, and once returning to Canada, and the potential impacts of their trip on third parties. This methodology may be replicated for the development of information sheets intending to communicate ethical concerns of other practices to providers or consumers of a certain service.

## Background

Medical tourism is the term commonly used in the industry to describe patients travelling across international boundaries with the intent of accessing non-emergency medical interventions [1, 2]. While historical accounts of persons receiving health care across borders exist, particularly in the case of patients travelling from developing to more developed countries for services that are not domestically available, a new trend appears to be emerging. Recent studies and media coverage indicate that patients from more developed countries in North America and Europe are intentionally leaving their countries of residence to access health care abroad [3, 4]. This health care is paid for out of pocket and may be motivated by long wait lists, unavailable medical procedures, and/or reduced costs for elective procedures requiring out-of-pocket payment domestically [4]. While patient travel across national borders to access medical care is assigned various terms in the academic literature, including international medical travel and transnational medical journeys, we use the term medical tourism in accordance with the term most commonly used in the industry [5, 6]. As Smith [7] points out, the term ‘medical tourism’ refers to a growing industry informing prospective patients about medical treatment options abroad to promote growth of the industry. Industry practices, compared to individual journeys for health care, produce distinct ethical concerns we discuss below related to patient safety and health system impacts if stakeholder decision-making is informed by sources with an interest in the profitability of the industry [7]. As it is these ethical concerns related specifically to industry practices that we are considering in this article, we use the term most commonly associated with these industry practices, ‘medical tourism’.

Media coverage of medical tourism has given attention to some of the practical dangers associated with this practice, including: surgical complications, risk of patients developing blood clots due to flying so soon after surgery, and potential for poor quality or unnecessary care due to a lack of regulation in the industry [8]. Increasingly, researchers have also discussed the ethical implications of medical tourism, such as potential negative impacts of health equity both within and between nations [9]. Research indicates, however, that medical tourists are often unaware of ethical implications and practical dangers associated with medical tourism [8]. Given medical tourists’ limited awareness of these ethical concerns, this paper describes and reflects on one possible response to this knowledge gap using an informational tool to prompt prospective Canadian medical tourists to consider ethical and safety concerns during their decision-making.

In the remainder of this paper, we describe our iterative process of developing an information sheet aimed at providing information to patients regarding ethical concerns of medical tourism that are not typically presented in industry and media sources of information. While this process was informed by the success of ethical buying guidelines in similar practices such as sustainable tourism, we use the term “information sheet” instead of “guideline” as there are limited regulatory bodies overseeing the industry who can develop and enforce activities that enable medical tourists to follow certain guidelines [4]. However, this information sheet intends to encourage contemplation and further public discussion of the ethical concerns in medical tourism to promote more ethical engagement in this practice.

### Ethical issues in medical tourism

Medical tourism may impact the health and well-being of individual patients as well as the general public of both destination and departure countries [10]. These impacts result in ethical concerns regarding the development of the medical tourism industry, in particular, concerns for patient health, and national and global public health [11]. Concerns for patient health include inadequate communication of risks to patients and failure to achieve informed consent [12]. These concerns are particularly relevant to the practice of medical tourism as there may exist certain challenges to communicating risk such as language barriers, time constraints of the medical tourism process, and/or financial interests of the medical tourism facility resulting in inadequate disclosure of risks [10, 12].

Discussions about ethical concerns of medical tourism have also focused on potential impacts on public health in both destination and departure countries. In destination countries, medical tourism may divert resources from the public to private sector, including health human resources and public funding [4]. Furthermore, the development of medical tourism industries may reorient the emphasis of public health in these countries on developing and providing health care which prioritizes the needs of international patients over domestic health needs [10]. This industry may also further privatize health care in these destinations, resulting in reduced access to health care for persons unable to pay [13, 14]. For countries of origin for medical tourists, there is concern that resources may be diverted from public health care to treat complications obtained from treatment abroad while persons leaving the country may reduce the pressure for health care system reform needed to meet the health needs of the population [11]. With this in mind, persons that are unable to travel for medical care may be unable to access needed health care through medical tourism [12, 13].

Finally, all of the ethical concerns for medical tourism have implications for global public health, particularly global public health equity [15, 16]. First of all, the movement of patients across borders may increase the transmission of infectious disease across borders [11]. The medical tourism industry may increase the provision of highly specialized medical treatment resulting in decreased provision of primary health care globally. This may result in increased global health inequity as health resources are concentrated in treating patients able to pay for specialized health care [4].

This summary of some of the primary ethical concerns related to medical tourism is not exhaustive and scholarship in this area continues to expand, including empirical research into the realized impacts of medical tourism [6]. Existing research shows that these concerns, particularly relating to third party impacts on members of the medical tourist’s home and destination countries, have not informed patient decision-making [4, 16]. In fact, patient health and safety experts in Canada have indicated a particular concern about uninformed decision making amongst Canadian medical tourists related to both safety and ethical considerations, indicating a need to better inform patients of these concerns [17]. It is for this reason that we have undertaken the development of an informational tool aimed at Canadians considering medical tourism.

### Ethical guidelines

Guidelines that inform individual decision-making exist in many domains with the aim of guiding persons’ actions to attain a result that is considered more ethical [18]. Ethical buying guidelines provide information on potential impacts of individual choices so consumers can make an informed decision while also creating an incentive for ethical practices by providers in response to consumer demands for more ethical goods and services [19]. Guidelines may support the development of social responsibility, which is the term used to describe the moral responsibility of an individual or group of individuals to promote the welfare of the communities to which they belong or with which they interact [11].

Globalization has resulted in the introduction of guidelines for social responsibility beyond borders, particularly corporate social responsibility for multinational corporations, as well as individual social responsibility for persons consuming goods and services beyond borders [20]. Sustainability is a core value for the development of many of these guidelines as this value encompasses environmental, social, and economic impacts of consumption [21]. Guidelines are commonly used as tools to promote sustainability by providing planners with goals and actions which contribute to sustainability and providing consumers with the know-how to purchase and act sustainably [22]. However, despite the increasing awareness of the role of public health and health equity in sustainability, guidelines for social responsibility are focused primarily on economic and environmental sustainability and less so on social sustainability, including use of health resources [23].

According to a collective ethical perspective, patients have a responsibility to use health resources efficiently and in ways that are beneficial to the community’s health [24]. Despite the existence of guidelines for social responsibility in practices involving other dimensions of sustainability, there is a concern about the lack of tools to support persons participating in activities that may impact upon global health equity [4]. As consumers of privately provided health care services, the development of tools to inform the decision making of medical tourists can draw on ethical values communicated in ethical guidelines that already exist for the consumption and use of goods and services in related areas, particularly ethical guidelines focused on achieving social sustainability.

While guidelines are used to promote social responsibility in sustainable tourism, medical voluntouring abroad, and the use of medical resources, all of which are global practices that involve the movement of people and/or resources between countries, medical tourists are provided with little or no guidance on their social responsibilities [25]. As global health equity for the industry are multifaceted and complicated by differing measures or indicators of health equity [26], a traditional ethical guideline that presents users with rigid rules for decision making is inappropriate. This is particularly evident from empirical accounts of medical tourism industry stakeholders differentially interacting with the industry according to sites of industry operation informed by different health system structures, regulatory and policy frameworks, and economic contexts [27–29]. Instead, we argue that medical tourists can benefit from a tool that provides information regarding a range of ethical issues associated with medical tourism that they can then use to consider how to engage in the industry as ethically and safely as possible in localized contexts of medical tourism industry pratices.

In this article, we describe the development of an informational tool that can be used to better inform the decision making of Canadians considering engaging in medical tourism with a focus on increasing their awareness of the practical dangers and ethical concerns associated with this global health service practice. Our development of this tool addresses a practical gap in the information available to potential medical tourists that has been identified by ethics and legal scholars in particular [24]. This activity was informed by existing ethical guidelines intended to promote social responsibility in the global community as well as emerging research on ethical issues surrounding medical tourism, including research highlighting the nuanced and complex contexts within which medical tourists participate in this industry. While the process described here is specific to an information tool for potential medical tourists, we believe this process can be meaningfully applied to other informational tools aiming to incorporate ethical considerations into decision making. This process will be particularly relevant to practices whose impacts on third parties are not well understood, as is the case with medical tourism presently.

## Methods

### Information sheet development methodology

**Fig. 1:**
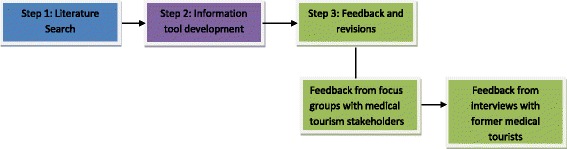
Multi-step process for information tool development

The research team underwent an iterative process to develop an information sheet intended to promote more ethical decision-making in medical tourism. Given that the majority of existing information for persons considering accessing medical care in the medical tourism industry are developed by sources with a vested interest in the profitability of the industry [24], this process was undertaken to develop a comprehensive and neutral source of information for prospective medical tourists. The development of this tool began with a review of existing literature regarding communicating information to promote socially responsible decision-making in consumption or service usage. The following section describes in detail the literature review, information sheet development, and subsequent revisions to the information sheet. Figure 1 illustrates the multi-step process that composes this methodology.

### Step one: Literature search

The development of an initial information tool for medical tourists took place through an iterative process that was informed by the use of existing ethical guidelines in areas that overlapped with the activities and concerns of medical tourists. Initially, we identified three relevant practices with established ethical guidelines, all representing one significant ethical domain of medical tourism. The selected practices include: tourism, the charitable provision of medical care abroad, and the use of domestic health care resources. A separate search for ethical guidelines was conducted for each of the three identified domains related to medical tourism.

To begin the search for guidelines, the databases Global Health, Medline, and Google Scholar were utilized. These databases were selected in consultation with a health sciences librarian and chosen with the aim of searching a wide variety of academic and policy sources and disciplines. As many guidelines are likely to be published by professional bodies, additional search strategies were also used to obtain a more comprehensive collection of guidelines for the selected practices. The additional search strategies for each topic of interest are explained in the following paragraphs.

The search for guidelines was conducted based on the team’s familiarity with academic and policy literature in the related areas. Search parameters included only English language documents identified using the search terms provided in Table 1 below. This process was done within a two-week period of time, at which point the research team decided there were sufficient guidelines to review to reach saturation of information. Overall, the research team found a large body of knowledge existing for sustainable tourism, as this is a growing and well developed practice aimed at developing long term solutions to mitigating stakeholder and environmental harms of tourism and sharing benefits among stakeholder groups [22, 30, 31]. Less information was available for medical volunteering and responsible use of health resources. However, by searching specific websites and venues throughout the English speaking world, this allowed for the inclusion of diverse guidelines which meet the inclusion criteria. Documents that were identified through this search process were reviewed if they included actionable recommendations to individuals considering participating in the activity.

**Table 1:** Selection of Guidelines for Literature Review

Guideline domains	Guideline Intention	Ethical concerns parallel to MT	Search terms utilized	Additional agencies searched
Sustainable Tourism	Encourage the use of local businesses and operations and activities that are environmentally friendly and culturally sensitive (Malloy & Fennel, 1998).	Concerns about negative impacts to the local economy, environment, and local culture due to tourist activities	‘Guide’, ‘frame’, ‘framework’, and ‘principle’ were each combined with the terms ‘sustainable tourism’, ‘social sustainability’, ‘economic sustainability’, and ‘ecotourism’	1) Caribbean Tourism Organization 2) Canadian Tourism Industry Association 3) Fair Trade in Tourism South Africa 4) The World Trade Organization 5) The Institute for Policy Studies 6) The Department for Communities and Local Organization 7) The Rainforest Alliance
Voluntourism	These guidelines are intended to guide health providers volunteering abroad to respect local standards, culture, and local access to health care (Provenzano, 2010)	Concerns about negative impacts of medical volunteers on sustainability of health care services, impacts on local standards, and impacts on local culture due to activities of volunteers	guide’, ‘frame’, ‘framework’, and ‘principle’ combined with ‘voluntourism’, ‘medical volunteering’, and ‘medical training abroad’	1) Medical school websites in Canada 2) Websites for professional bodies including colleges of physicians and medical associations from Canada, the US, Australia, New Zealand, and the UK.
Use of publically funded health resources	These guidelines are intended to encourage responsible use of finite health resources for individual patients and health care providers (Smith, 2002; Commission on the Future of Health Care in Canada, 2002)	Concerns about negative impacts of individual health resource consumption on equitable distribution of health resources	‘guide’, ‘frame’, ‘framework’, and ‘principle’ with the terms ‘patient responsibility’, and ‘patient use of health care’	1) Canadian provincial and national level health ministry websites 2) National health association and ministry websites in the US, Australia, New Zealand, and the UK. 1) U.S. websites for Medicaid and Medicare

### Step two: Information tool development

Once the initial search for guidelines had been completed, we had accumulated a total of 35 guidelines (18 guidelines on sustainable tourism, 11 guidelines on medical volunteering, and 6 guidelines on the responsible use of health care). After compiling reference details for the guidelines into a spreadsheet, each was reviewed by two members of the research team. Information was also independently recorded in the spreadsheet from each team member on the intended audience of the guideline, values promoted by the guideline, format of the guideline, and themes of the guideline. After completing this activity, we reviewed the spreadsheet and then met to discuss important findings from this activity. From this meeting, we agreed to focus first on identifying common ethical values that emerged from the various guidelines that would be relevant to ethically engaging in medical tourism. Our purpose in identifying ethical values was to inform the recommendations provided to medical tourists with these values to ensure the development of a comprehensive tool. These values were independently identified by each team member and then were discussed in a meeting, after which consensus was reached on 9 values including: sustainability, efficiency, stewardship, equity, reciprocity, non-maleficence, autonomy, empathy, procedural justice.

Following identification of the 9 values that were commonly included in the 35 reviewed guidelines, we next met to discuss how to use these values to inform the development of a tool that could successfully inform potential Canadian medical tourists about ethical issues with medical tourism. At this point it was agreed that we needed to shift these ‘high level’ ethical values into precise and actionable recommendations for intended informational tool users. We determined that sets of both goals and actions should be included on the tool. The goals would provide overall desired indicators of an ethical medical tourism industry drawn from all 9 of the values. The list of actions would be drawn from the values and goals but would be more specific by providing examples of actions that would help achieve the desired goals.

To create goals that would inform the actions of each stakeholder, we identified stakeholder groups that could contribute to the realization of a more ethical medical tourism industry. The identification of stakeholders and involvement of various stakeholders in tourism planning and development is considered a necessity to ensuring sustainable tourism industries and practices [32]. Using previous knowledge of the literature on medical tourism, we identified three stakeholder groups as having the most influence over an ethical medical tourism industry: patients engaging in medical tourism, providers of medical tourism services, and members of the destination communities. These stakeholders were identified as each having control over broad domains of the practice of medical tourism including individual patient choices, provision of medical services aboard, and governance of the provision of medical services abroad.

With this set of stakeholders in mind, we next underwent the process of developing goals and actions to populate an informational tool for the development of ethical medical tourism for each of these stakeholder groups. Using information gained through the process of reviewing the guidelines in related practices and familiarity with the literature on medical tourism, based on the ethical values established we independently created a list of goals for each stakeholder group. This list of goals was meant to depict medical tourism practices that address ethical concerns with the impacts of medical tourism and is reflective to the full list of values for all stakeholder groups. Through a process of review and consensus, 15 goals were selected that were determined to represent all elements of the objective of creating a more ethical practice of medical tourism across the three stakeholder groups.

Once the list of goals had been determined, we met again to establish actions that could help achieve these goals. We focused on each stakeholder group individually and developed categories of actions that would address the 15 goals. As each stakeholder group has a distinct range of authority and influence, it was felt that the actions for each group should be distinguished from one another. We individually created a list of possible actions for each stakeholder group, and listed the goals that informed each action. These actions were developed in response to the goals but were not intended to represent a one-to-one correlation with each goal. After discussing all of the suggested actions, we established a final list of actions for each stakeholder group that synthesized the lists from each team member.

The list of values, goals and actions were reviewed once more in a collaborative meeting in order to make final edits to the wording and inclusion of values, goals, and actions. While editing, we decided to identify one primary goal that informed each action, whereas multiple goals of varying relevance had previously been identified. We went through the action lists and agreed via consensus on the primary goal informing each action. At the end of this activity, we verified that all goals were represented by an action and eliminated any goals that did not inform the finalized lists of actions. This resulted in the elimination of 2 goals (maintains *confidentiality* for international patients; recognizes the *global interconnectedness* of the medical tourism industry) to create a final list of 13 goals. Table 2 below provides the finalized list of goals.

**Table 2:** List of goals

1. is *adaptable* to local and global changes;
2. promotes *capacity building* in the public and private health sectors;
3. *promotes and maintains* the health of international patients and local populations;
4. has *consideration* for the wellbeing and needs of local populations and international patients;
5. is consistent with local *cultural values* and exhibits cultural awareness for members of local populations;
6. *distributes* the benefits created by this sector fairly;
7. *protects* all stakeholder groups from new harms;
8. promotes and *empowers* informed decision making by international patients;
9. promotes *collaborative decision making* by local stakeholders;
10. is *responsive to power imbalances* among stakeholders in the development of the sector and distribution of its benefits;
11. protects and enables the *integrity and dignity* of all stakeholders;
12. exhibits *shared responsibility* among stakeholders for the impacts of the sector; and
13. promotes *transparency* in decision making around the development, administration, and impacts of the sector.

Using the finalized lists of values, goals, and actions, a first draft of the information tool was created. The format of this draft tool was informed by a template of a guideline for sustainable tourism developed by the Tourism Industry Association of Canada [29]. This structure was identified as a good format in comparison to other guidelines that were reviewed as its structure and layout clearly demonstrates the specific actions that can contribute to ethical medical tourism, as well as informs users of the underlying values that inform these actions. We also incorporated examples paired to each action as part of the draft tool to better inform stakeholders on potential impacts of medical tourism. Each action was coupled with both general examples that would apply in all situations, as well as a more context-specific example to help illustrate the action described. All members of the research team reviewed the draft tool and made any necessary revisions to create a finalized draft tool.

### Step three: Feedback and revisions

According to social marketing methods, formative research is a critical component in the development of public health interventions that advocate for behavior change [33]. Formative research includes pretesting concepts and materials with the intended audience and/or gatekeepers to this audience [25]. To begin this formative research stage, we conducted three focus groups with medical tourism stakeholders. The cities of Vancouver, Montreal, and Toronto were selected as focus group sites as they are all large and diverse Canadian metropolitan centres that have significant numbers of local members who are active in the medical tourism industry and Canadian public health care activities, while representing geographic, cultural, linguistic and economic diversity within Canada. The purpose of these focus groups was to allow for feedback on the draft tool created in Step Two from stakeholders across Canada. We identified relevant stakeholder groups including public health representatives, health authority officials, family physicians, former medical tourists, and patient advocates. Participants from each of these groups were invited to attend the focus groups.

We developed an agenda for facilitating the focus groups, which included an overview of the meeting goals, a background to medical tourism and key ethical issues in the medical tourism literature, followed by an open discussion around three questions: 1) What are the anticipated ethical concerns created by medical tourism; 2) Based on your previous review of the draft informational tool, what additions, alterations, and cuts should be made to its content; and 3) What means are available for informing potential medical tourists about these ethical issues and for most effectively distributing this tool? The focus groups lasted 2 h each and were recorded for verbatim transcription. Notes were also taken by one of the members of the research team. Eight stakeholders participated in each of the Montreal and Toronto events, while nine participated in Vancouver.

We reviewed the transcripts from these focus groups and identified six primary recommended changes to the informational tool according to the expert opinions of the focus group participants. These recommendations included: 1) shortening the tool; 2) developing a background section on ethical concerns; 3) removing the list of values; 4) providing more practical sample questions and using a checklist format; 5) developing a webpage for further information; and 6) using language that clearly does not appear to endorse medical tourism. With these recommendations in mind, we discussed strategies for revising the tool to fit with the feedback provided by focus group participants while still meeting the aim of the tool itself and acknowledging limitations in time, resources, and data. At this point in the revision process, we agreed that a traditional guideline document that guided the decision-making process of potential medical tourists would be impractical given the dearth of data on the impacts of medical tourism on stakeholders as well as the complex contexts within which medical tourists decide to travel [6]. For this reason, we narrowed our focus on an information tool that would raise awareness for users of the potential of medical tourism to create ethical problems, among other issues. As a result, we shortened the draft tool, eliminating the list of values, created three separate sections for ease of use, and adjusted the language so that it reads more like a checklist with prompts for potential medical tourists to consider a variety of potential impacts of medical tourism, both on individual safety and health systems. The final information sheet differed from our initial draft versions in its intended aim; instead of trying to prompt particular actions, stakeholder feedback suggested that with limited regulatory oversight of the industry and time and resource constraints limiting prospective medical tourists’ decision-making, the information sheet should aim instead to stimulate conversation about the topic.

**Fig. 2:**
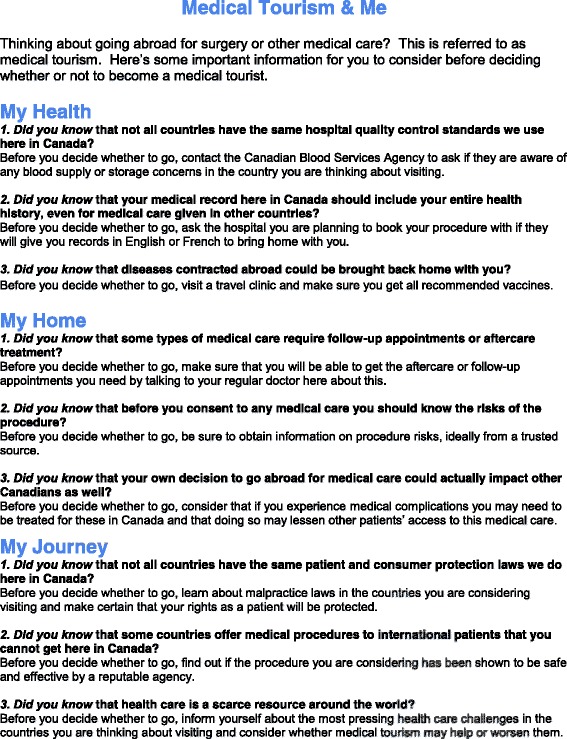
Draft information sheet

Following the completion of the revisions to the informational tool, we pre-tested the tool (as seen in Fig. 2) with the intended users of this tool by conducting semi-structured interviews with Canadians who had already opted to engage in medical tourism [30]. As the target audience includes all Canadians that may be considering travelling out of country for care or do not intend immediate action but may contemplate such a decision in the future, we developed a tool that aims to inform not persuade [34]. Since the target audience is difficult to identify, we pretested our tool with Canadians that have already travelled out of country for medical care. This participant group was well positioned to provide insight on the usefulness of such a tool having already engaged in decision-making around opting to seek private medical care abroad [30].

Recruitment of interviewees through Craigslist postings, Facebook postings on patient advocacy groups, advertisement in a local free weekly newspaper, as well as contact information in newspaper articles about medical tourism resulted in 24 interview participants. Recruitment and interviewing occurred simultaneously until a predetermined termination date. Before beginning the interview, participants confirmed their eligibility for the study, were provided with details about the study, and provided consent to participate. Eligibility criteria for the study required participants to be: 1) 18 years or older; 2) a holder of a provincial medical card; 3) someone who successfully pursued a surgical procedure outside of Canada that was neither a transplantation nor a reproductive surgery (as these procedures often involve third parties and raise distinct ethical issues); and 4) someone who paid privately for the surgery sought abroad and for whom the procedure was not performed based on a referral from a Canadian physician.

The first author performed 23 of the interviews, while the second author performed 1. All interviews were recorded and transcribed verbatim. Interviews lasted on average 45 min and interviewers asked questions from a semi-structured interview guide. This guide had 4 components: the first component consisted of basic background demographics, followed by a section on background information on decision-making, a section regarding the decision-making process of deciding to travel for medical care, and finally a section with questions asking for feedback on the draft informational tool. All participants were provided with a copy of the information tool prior to the interview and were instructed to read it in advance as well as have a copy on hand during the interview to provide as detailed of feedback as possible.

After all of the interviews were completed, transcripts were read in their entirety by the first author. Three transcripts selected as being a representative sample of positive and negative feedback on the informational tool were read in full by all group members. Each member was also assigned three unique transcripts to read, once again representing a range of feedback on the tool as well as a range of medical procedures. Following independent transcript review, we met to discuss common themes emerging from the interview participants and implications for this feedback for subsequent revisions to the tool. To ease this process, sections of the transcript specifically dealing with feedback on the tool and recommendations for changes to the tool were extracted by the first author and compiled into one document with positive and negative feedback divided into separate sections. This document was also read independently by all members of the research team.

**Fig. 3:**
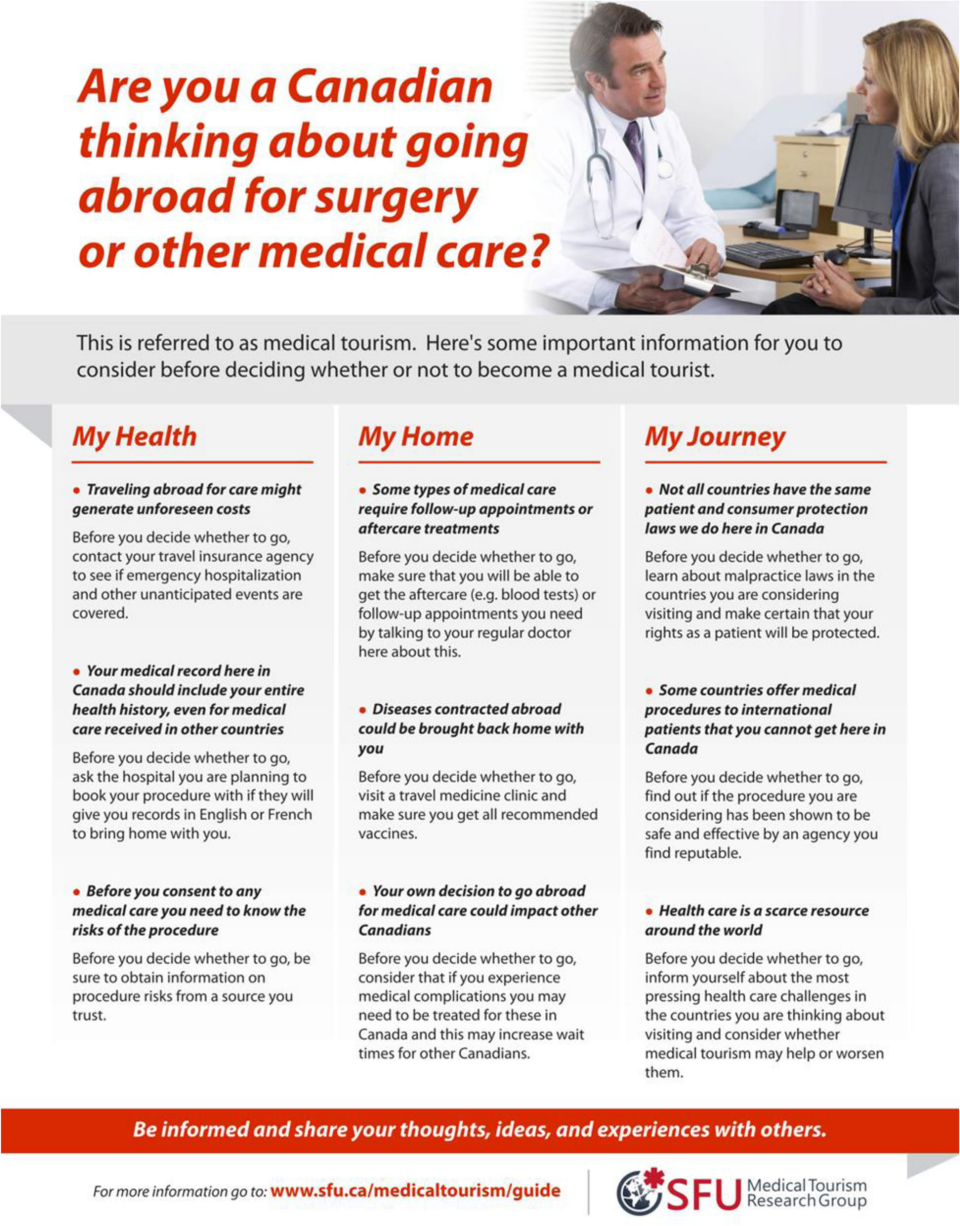
Final information sheet

Following the above-mentioned steps around transcript review, the research team focused on addressing the recommendations provided by medical tourism stakeholders, including recommendations related to content and formatting. Content recommendations emphasized participant frustrations. To address formatting suggestions, we once again consulted published materials to identify formatting strategies for communicating desired information. In this case, we focused on collecting health information tools from travel clinics and doctors’ offices to see what formats are successful in these intended dissemination locations. Formatting ideas from these existing tools were taken into consideration, including: 1) Using bold words and colour effectively to enhance key messages; and 2) Minimizing extra or repetitive wording. Multiple formatting samples were created and were shared within our networks for feedback to ultimately determine a final format of the information sheet, as seen in Fig. 3. We disseminated the final information sheet to travel clinics, primary care physicians, and through social media.

## Discussion

Medical tourism is an emerging global health practice and despite its growing popularity there is a significant dearth of third party, or non-industry-driven, information available for patients considering purchasing such. In response, we sought to create in informational tool for Canadians considering medical tourism that would inform them of things they should give attention to before purchasing private medical care abroad. Some patient-specific informational tools for medical tourism do exist for specific procedure types. For example, the International Society for Stem Cell Research has developed a list of key questions for patients considering engaging in stem cell tourism [35]. These eight questions focus on raising patient awareness about safety issues tied to unproven stem cell technologies and increasing credulity about claims made by industry members, especially around success rates for and the safety of these treatments. Similarly, the American Society of Plastic Surgeons lists questions for patients considering traveling abroad for care that focus on obtaining follow up care, protecting patient safety, and encouraging more critical approaches to surgeons’ claims [36]. A more general patient guide produced by the International Medical Travel Journal advises patients on decision-making, finding a clinic to suit their needs, locating aftercare, and patient safety issues [37]. The concerns raised in these existing tools are much narrower than in our own, focusing on the impacts of medical tourism on the patient rather than other stakeholders. Thus, the method for developing the information tool described here fills an important gap in the limited set of resources available and helps to raise awareness of the impacts of medical tourism on a wider range of stakeholders. Our tool is also meant to be consumed by prospective medical tourists traveling for a variety of medical procedures, limiting our ability to provide actionable guidelines as we learned throughout this process.

Overall, our approach to developing an information tool for informing patients about the ethical dimensions of engaging in medical tourism includes three distinct advantages. First, this method allows the development of a tool that draws from the content, structure, and diversity of guidelines in other, related practices. In this way, the process of developing a new information tool for a practice does not ignore but rather incorporates successful models developed elsewhere. Rather than simply parroting the concerns raised within the literature on the practice for which the information sheet is being developed, this method ensures that the scope of values, goals, and actions is broadened in light of the experiences in other practices. This method is particularly helpful for a relatively new and developing practice such as medical tourism where discussion of the ethical dimensions of this practice is still evolving and a consensus regarding responses to these concerns has not been reached [10, 11].

Furthermore, by looking to ethical guidelines in related practices to first identify the values that informed our tool, the development of this information tool is grounded in theories of sustainability [38]. While the concept of “sustainability” is not clearly defined, it is used in this case to describe the outcome of collective agreement on current and future livelihoods, with a focus on social justice. Research in the area of sustainability has indicated a need for methods of deliberation that engage communities and individuals in contemplating and deciding on the future they want to create as the core component to attaining sustainability [39]. By drawing on ethical guidelines that engage with the values of sustainability, this tool encourages similar processes of deliberation in consumer decision-making and industry engagement. While we recognize that this deliberation might fail to consider ethical concerns specific to particular medical tourism practices, we argue that the deliberation encouraged by this information sheet enables context specific and even invisible ethical concerns of medical tourism practices to come to light through public engagement in this deliberation [40, 41]. Deliberative public engagement could highlight new areas of ethical concern through discussions of systemic reasons for traveling abroad for health care [41].

Second, the collaborative methodology used here ensures that no single voice or perspective has dominated in development of the information tool. While each of us completed reviews of other guidelines and initial lists of values, goals, and actions independently, the collaborative process of comparing lists and finalizing a draft tool was extremely important for improving the quality and usefulness of the final product. We initially felt that face-to-face time for this process would be minimal, but ultimately found that settling on a structure for the information tool and final content required extensive discussion – in our case well over 12 h of discussion over five meetings, not including follow-up discussion via email. We strongly believe that this collaborative method is an important advantage and irreplaceable aspect of the tool design process we developed. Furthermore, by utilizing both focus groups and interviews with different target audiences, multiple perspectives informed the revisions to this tool. Participants of the focus groups provided useful feedback from an audience that regularly provides health information and would potentially be involved in the dissemination of such a product, otherwise known as the gatekeepers in health communication literature [30]. Interview participants provided useful feedback from the intended audience and encouraged further revisions to enhance the appeal and utility of such a tool. By utilizing methods found in health communication literature including social marketing, this methodology maximizes the effectiveness of the tool as a public health intervention [42].

Finally, the methodology described here can be easily adapted to other practices. As the choice of other practice domains to review was based on locating practices that overlapped with significant aspects of medical tourism, other domains can be determined for other practices. In this way, new information tools can be developed in a way that challenges the assumptions of that practice through reviewing other, related practices and through a collaborative methodology. These advantages should accrue both for relatively new practices like medical tourism that has very few existing guidelines for any stakeholder groups, but it can also be used to challenge the assumptions and develop new sources of information for consumers of practices with more established guidelines as well.

### Limitations

This information tool development method does face several limitations, however. First, while the collaborative nature of the development process ensures that the viewpoint of a single team member does not dominate the content of the tool, in this case we had been collaborating with one another on research projects related to medical tourism and co-authoring papers for over 2 years. While this existing relationship did not mean that we would share the same perspective in the tool development process, particularly as we represent distinct disciplinary backgrounds, it does imply a degree of insularity to the development process and reduces the range of perspectives and challenging voices that will be heard. While others using this method may not face this limitation, it is likely that other well-established research teams will use this process as the team must be familiar with the literature on the practice for which the tool is being developed in order to successfully populate the tool. Presentation of draft versions of the guide to other stakeholders helps to ensure that any insularity in the core tool development group will be overcome. While doing so increases the time and financial resources needed to develop an information tool, we feel strongly that it greatly enhanced the quality and usability of the final product.

A second limitation of the methodology described here is the uncertainty of whether the tool conveys the ethical values that were originally intended to be addressed in this document. As mentioned, interview participants expressed frustration about either information that was irrelevant to their experiences or the lack of actionable recommendations in the tool, indicating that the information may not be taken into further consideration as intended. The purpose of the tool is to provide information that prompts further deliberation and research by individuals when considering accessing medical care outside of Canada; however, it is unclear from the interviews whether the statements provided in the tool accomplish this aim. We were challenged in developing this guideline to try and produce a pragmatic document that could be used by prospective medical tourists traveling for a variety reasons while trying to capture ethical and safety concerns relevant to unique individual experiences so as to not disengage readers who feel the content does not speak to their experiences. For example, when engaging in discussion about ethical concerns, interview participants often focused on what they perceived as the unethical and inequitable nature of the Canadian health care system and how this pushed them into obtaining medical care abroad. However, the frustrations of interview participants also expanded our discussions within the focus groups and interviews to ethical and safety concerns we had not identified, suggesting this informational tool could also stimulate reflection and dialogue about engagement with the industry. This process aligns with literature on deliberative bioethics which indicate that public engagement with ethical considerations can allow nuanced and invisible experiences to inform more ethical engagement in practices such as health care [41].

Finally, as our focus is on tool development, we have not addressed the issue of dissemination in detail here, which may be a particular challenge with our target audience [30]. While potential medical tourists have been found to engage in research on the internet prior to engaging in medical tourism [43, 44], we anticipate that it will be difficult to ensure the visibility of any new information tool amidst the many competing sources of information, particularly those in the medical tourism industry. According to social marketing methods, dissemination of health communication materials requires substantial consideration of barriers to use, recognition of benefits by the target audience, appropriate location for dissemination, as well as promotional strategies of the information tool [42]. This process requires further time and resources to ensure effective dissemination and use. Additionally, this requires buy-in by stakeholders to support the dissemination of this tool. Our own future steps are to develop a dissemination strategy for the tool that is consistent with stakeholder feedback, to create a complementary website for the tool where additional information can be shared, and to develop an evaluation strategy both for uptake of the tool as well as its utility.

## Conclusions

This paper responds to a gap in existing methodology for developing ethical guidelines for healthcare consumers. As such, this paper explores the development of one such collaborative methodology. By describing the steps and considerations of developing the information sheet, including a literature review, group meetings to develop the tool, and feedback and revisions from medical tourism stakeholders as well as the strengths and limitations of this process, this paper provides a discussion of the ability for such a methodology to develop an effective information tool for communicating ethical concerns.

While the tool development process outlined in this paper has focused on the issue of medical tourism, we contend that there is wider potential use for this process so as to generate information tools for other distinct practices. This is particularly true for other practices where information about its impact on third parties is limited such as with environmental impacts on health. The process outlined herein can be adopted fully in such cases or adapted so as to be more relevant to the practice at hand. In either case we encourage researchers to document the process by which they have developed their informational tools so as to enhance transparency about the research-to-action cycle around the creation of ethics-informed resources and guidelines.

The practice of engaging in research and shifting research into action often has unanticipated benefits. In our case, we were surprised both by the degree to which this tool development process challenged our own assumptions about the preferred shape of the tool being developed and the degree to which continued discussion was needed to arrive at consensus about the final content of the tool, including discussion with stakeholders outside the research realm. We see this as a significant strength of having undertaken the process outlined in this paper.
